# Oxacillin (Methicillin) Resistant Staphylococci in Domestic Animals in the Czech Republic

**DOI:** 10.3390/pathogens10121585

**Published:** 2021-12-06

**Authors:** Jaroslav Bzdil, Monika Zouharova, Katerina Nedbalcova, Vladimir Sladecek, David Senk, Ondrej Holy

**Affiliations:** 1 Ptacy s.r.o., Valasska Bystrice 194, 75627 Valasska Bystrice, Czech Republic; vetmed@seznam.cz (J.B.); sladecek.vladimir@gmail.com (V.S.); ptacy@ptacy-sro.cz (D.S.); 2 Department of Infectious Diseases and Preventive Medicine, Veterinary Research Institute Brno, Hudcova 296/70, 62100 Brno, Czech Republic; monika.zouharova@vri.cz (M.Z.); katerina.nedbalcova@vri.cz (K.N.); 3 Science and Research Centre, Faculty of Health Sciences, Palacky University Olomouc, Hnevotinska 3, 77515 Olomouc, Czech Republic

**Keywords:** prevalence, occurrence, organs, virulence genes, susceptibility, veterinary medicine

## Abstract

The aim of this study was to describe the prevalence of different *Staphylococcus* species isolated from pathological processes and lesions in domestic animals in the Czech Republic and to detect and describe oxacillin (methicillin)-resistant strains (MRS). During the years 2019–2020, a total of 5218 veterinary clinical samples from the Czech Republic were tested. Testing was performed by culture methods and typing by molecular phenotypic methods MALDI-TOF MS and PCR. Antimicrobial susceptibility testing of the strains was performed by the disk diffusion method. A total of 854 staphylococci strains were identified (16.37% prevalence), out of which 43 strains of 6 species of staphylococci were MRS (*n* = 43; 0.82% prevalence). Of the MRS strains, the most prevalent species were *Staphylococcus pseudintermedius* (*n* = 24; 0.46% prevalence) and *Staphylococcus aureus* (*n* = 7; 0.13% prevalence). Susceptibility testing showed resistance to beta-lactam antibiotics and, depending on the species, also to trimethoprim/sulfamethoxazole, gentamicin, tetracycline, erythromycin, clindamycin, and enrofloxacin. For further characterization of MRS, PCR assay for virulence factor genes was performed. Seven of the 14 target genes were observed only in *S. aureus*, except for the *eno* gene encoding laminin-binding protein, which was also detected in other staphylococci. It is necessary to emphasize the issue of correct using of antimicrobials in practice and antibiotic policy in university teaching and to create stricter legislation that would prevent the widespread use of antimicrobials in veterinary medicine, especially in livestock to reduce the emergence and spread of antimicrobial resistance.

## 1. Introduction

Several studies have shown that Gram-positive bacteria are apparently the most common microorganisms isolated from different human and veterinary clinical materials [1,2,3,4,5]. This group of bacteria includes staphylococci, which are mostly commensals of the skin and mucous membranes in animals and humans [6,7]. Many of them are opportunistic pathogens causing pyogenic infections [6,8]. Of the coagulase-positive staphylococci (CPS), the species of special human and veterinary importance is *Staphylococcus aureus*, which causes local purulent and systemic infections, as well as human and animal toxaemia [5,9,10,11]. Other CPS such as *Staphylococcus pseudintermedius*, *Staphylococcus intermedius*, and *Staphylococcus delphini*, which are included in the *Staphylococcus intermedius*-group, as well as *Staphylococcus schleiferi* ssp. *coagulans* and *S. hyicus*, are significant primarily in the veterinary sphere [6,12,13,14].

Coagulase-negative staphylococci (CNS) have been considered a component of normal microbiota but can also act as human and animal pathogens due to their invasiveness, production of toxins [6,13], adhesins and hemolysins, and the ability to form biofilms [1,3,7]. Members of this group can cause a wide range of animal and human diseases, namely, local infections of skin, mucous membranes of urinary and respiratory apparatus, and mammary gland, and they have been reported as a potential cause of septicaemia in human and animals [2,9,13,15,16]. Due to biofilm formation, some CNS strains (for example, *S. epidermidis*) can also cause foreign body-associated infections [17]. Some *Staphylococcus haemolyticus* strains can cause infections in debilitated dialysis patients, diabetic patients, and patients after surgery, and it is well known for its ability to develop multidrug-resistant forms in bedridden patients [18]. Staphylococci as a zoonotic agent and a source of resistance and pathogenicity genes for people are a current topic of the present time. Many recent works have described different cases of human infections caused by animal strains of staphylococci or food-born staphylococci strains [8,11,19,20,21]. 

Varying levels of antimicrobial resistance are found in human and veterinary strains of staphylococci. Regarding therapy, methicillin-resistant strains of staphylococci need special attention because they show co-resistance to other beta-lactam antibiotics including oxacillin/methicillin and, in many cases, also to aminoglycosides, tetracyclines, macrolides, chloramphenicol, fluoroquinolones, and rifampicin [9]. 

Oxacillin resistance is encoded by the *mecA* gene [9] and its two homologues, mecB and mecC, with several alotypes. As known for mecA, the gene homolog mecC is also not restricted to *S. aureus*, but found in several staphylococcal species including *S. sciuri*, *S. stepanovicii*, and *S. xylosus* (mecC1 allotype). First investigations showed a wide geographical distribution of mecC-MRSA in Europe and a broad diversity of host species including livestock, companion, and wildlife animals. In particular, wild rodents and insectivores might serve as a reservoir for staphylococci harboring mecC [22]. Despite the fact that methicillin resistance has no particular effect on the clinical course of disease, it can fundamentally affect the antimicrobial therapeutic effect and, even worse, may be a source of resistance genes for other *S. aureus* strains in other animals and humans [23]. In human medicine, methicillin-resistant *S. aureus* (MRSA) is often detected among the hospital-associated methicillin resistant *Staphylococcus aureus* (HA-MRSA) strains [9] and, in veterinary medicine, staphylococcal resistance to methicillin (oxacillin) was detected, particularly in cows with mastitis or in skin lesion samples from cats and dogs [12,23,24,25]. 

Staphylococci can express a wide range of virulence factors including surface proteins, exoenzymes, and extracellular toxins that allow it to adhere to biotic or abiotic surfaces, invade or avoid the immune system, and cause harmful toxic effects to the host. Ownership of these factors may strongly influence the course and severity of infection [26]. 

The first step of infection is bacterial adhesion to host extracellular matrix and plasma proteins. It is mediated by different proteins of the family of MSCRAMMs (microbial surface components recognizing adhesive matrix molecules) [27]. These molecules include *EbpS* (elastin-binding protein), *Eno* (laminin-binding protein), *Cna* (collagen-binding protein), *Fib* (fibrinogen-binding protein), and *Bbp* (bone sialoprotein-binding protein). The *FnbA* and *FnbB* proteins bind to fibronectin and fibrinogen, while clumping factors *ClfA* and *ClfB* bind to fibrinogen and promote bacterial adhesion to thrombi. Binding of these surface proteins to various substances present in the extracellular matrix allows the bacterium to invade host tissues [28]. 

Another very important step is accumulation of bacteria in multi-layered cell clusters. Such biofilm formation protects microorganisms from opsonophagocytosis and antimicrobial agents [29]. Biofilm formation in isolates occurs through the polysaccharide intercellular adhesin (PIA). The intracellular adhesion (*ica*) operon is essential for the control of biofilm production. The *icaADBC* operon encodes three membrane proteins (IcaA, IcaD, and IcaC) with enzymatic activity and one extracellular protein (IcaB). The PIA, encoded by this operon also plays an important role in adhesion to epithelial cells and allows for escaping the immune system of the host [29]. 

Staphylococci may have a number of other virulence factors that are involved in the pathogenesis of the disease. Some symptoms associated with *S. aureus* infection are caused by toxins, such as toxic shock syndrome toxin 1 (TSST-1), enterotoxins, and exfoliative toxins (ETs). More than 20 SEs have been identified to date and are one of the most frequent causes of food-borne disease. TSST-1 causes a serious illness with high mortality. Exfoliative toxins (ETs) (also known as “epidermolytic” toxins) cause staphylococcal scalded skin syndrome (SSSS) characterized by destruction of desmosomal cell attachments resulting in detachment of the epidermis [9,30] (Votava et al. 2006; Oliveira et al. 2018). Some strains of *S. aureus* can also produce Panton–Valentine leucocidin [4,16]. 

The presence of virulence factors in different staphylococcal strains varies depending on the location of the infection and the degree of virulence.

The aim of the present study was to describe species distribution of staphylococci isolated from pathological processes and lesions in domestic animals in the Czech Republic and to detect and characterize oxacillin/methicillin-resistant staphylococcal (MRS) strains, including their prevalence, site of infection, host specificity, and virulence factor determination.

## 2. Material and Methods

### 2.1. Isolation and Identification of Bacteria: Bacteriological Confirmation

A total of 5218 clinical samples from pathological processes and lesions of domestic animals between April 2019 and June 2020 underwent microbiological cultivation at the Veterinary Research Institute Brno (Czech Republic). Sampling was performed by instructed private veterinarians. Solid and slurry materials were sterile collected in 60 mL plastic containers (Dispolab Ltd. Brno, Czech Republic). Fluids were collected in 10 mL sterile closable tubes (Dispolab Ltd. Brno, Czech Republic). Swabs were taken using Transbak system containing Amies broth with active carbon (Dispolab Ltd. Brno, Czech Republic). All samples were stored and transported to the laboratory at 4 °C.

### 2.2. Samples from Digestive Tract

Feces, rectal swabs, and swabs taken from the stomach lining were examined routinely by conventional methods of cultivation on meat peptone blood agar (MPBA) (Trios Ltd., Prague, Czech Republic), and plates were incubated aerobically at 37 ± 1° C for 24 h. 

### 2.3. Samples from the Skin; Urinary Apparatus; Oral Cavity; Eyes; Respiratory, Musculoskeletal and Lymphatic Systems

The cultivation of hair; swabs and scrapings of skin; swabs of ear; urine and swabs of the urinary tract; swabs and the lavages of the respiratory tract, pharynx, conjunctiva and oral mucosa; the puncture of chest, lymph nodes and joints were performed on MPBA (Trios Ltd. Prague, Czech Republic), and the plates were again incubated aerobically at 37 ± 1° C for 24 h.

### 2.4. Mammary Gland and Milk Samples

The milk samples were again inoculated on MPBA (Trios Ltd., Prague, Czech Republic) after thorough shaking, and incubation was carried out aerobically at 37 ± 1 °C for 42–48 h.

### 2.5. Bacteriological Confirmation and Susceptibility Determination

All types of colonies grown on plates were isolated, and suspected Gram-positive organisms were isolated and subsequently confirmed by the phenotypic molecular method using mass detector MALDI-TOF MS Microflex^TM^LT System (Bruker Daltonik GmbH, Bremen, Germany), on the basis of proteomics analyses and MALDI Biotyper software MBT Compass 4.1.100 (Bruker Daltonik GmbH, Bremen, Germany). In the plates with mixed bacterial cultures, the most prominent colony-forming unit agent was regarded as the leading pathogen. An identification score of 2.000 was set as the reliability threshold. The typing of strains with a lower score was specified by the MALDI-TOF method using a more exact library of spectra of the National Reference Laboratory for Staphylococci of the National Institute of Public Health in Prague or PCR method.

### 2.6. Antimicrobial Susceptibility Testing

Clinical strains were tested for antibiotics susceptibility by the disc diffusion method. The Mueller–Hinton agar (Trios Ltd., Prague, Czech Republic) and antibiotic discs were used for testing (Oxoid Ltd., Basingstoke, United Kingdom). The tested antibiotics were shown in Table 1. The tests were assessed after 18–24 h of incubation at 37 ± 1 °C. The interpretation of values according to CLSI (2013), CLSI (2018), NCCLS (2002), EUCAST (2020), CASFM (2018), BD BBL (2020), and BIOPHARM (2020) standards was performed (see the Table 1) [31,32,33,34,35,36,37]. The quality control of used discs and media was performed with reference strains *Escherichia coli* (ATCC 25922) and *S. aureus* (ATCC 25923).

### 2.7. MRS Molecular Characterization

In 35 MRS isolates including *S. pseudintermedius* (*n* = 20), *S. aureus* (*n* = 7), *S. haemolyticus* (*n* = 4), *S. intermedius* (*n* = 2), and *S. epidermidis* (*n* = 2), polymerase chain reaction (PCR) for *mecA* gene detection was used to confirm methicillin resistance. Eight strains failed to resuscitate, and PCR was not performed. Gene *mecA* encodes the production of penicillin-binding protein PBP2A (or PBP2’) and is considered the gold standard for methicillin resistance determination. For characterization of MRS isolates, the presence of virulence factor genes, including MSCRAMM, biofilm and the main toxin genes, were detected by PCR (polymerase chain reaction). The following genes were targeted: *cna* (encoding collagen-binding protein), *eno* (encoding laminin-binding protein), *clfA* and *clfB* (encoding clumping factors A and B), *fib* (encoding fibrinogen-binding protein), *ebp* (encoding elastin-binding protein), *bbp* (encoding bone sialoprotein-binding protein), *fnbA* and *fnbB* (encoding fibronectin-binding proteins A and B), *icaA* (encoding polysaccharide intercellular adhesin), *etA* (exfoliative toxin A), *etB* (exfoliative toxin B), and *tsst* (encoding toxic shock syndrometoxin) (see Table 2). 

Quick boiling method was used for DNA isolation. A number of colonies of pure bacterial culture were resuspended in 50 µL of sterile distilled water. The suspension was incubated for 10 min at 100 °C and centrifuged for 10 min at 10,000× *g* at 4 °C. The supernatant was used in the PCR reaction as template DNA. Polymerase chain reactions were performed according to the protocols described in references in Table 2. Fragments were analyzed by electrophoresis in 2% agarose gel stained with ethidium bromide and visualized under ultraviolet light. Strains used as positive controls were *S. aureus* CCM 2353 for *cna*, *eno*, *clfA*, *clfB*, *fib*, *ebp*, *bbp*, *fnbA*, *icaA*; *S. aureus* CCM 2773 for *fnbB*; *S. aureus* CCM 7056 for *etA* and *etB*; and *S. aureus* RF122 (Fitzgerald J.R., University of Edinburgh, Edinburgh) [43] for *tsst* gene.

## 3. Results

Out of 854 (16.37% prevalence) isolated staphylococci strains, 613 strains belonged to the group of CPSs (11.75% prevalence) and 241 strains to the group of CNSs (4.62% prevalence). The detailed numbers and prevalence of different species of staphylococci are shown in Table 3. Of these, 43 strains of 6 species were methicillin-resistant staphylococci (MRS) (*n* = 43; 0.82% prevalence). Of them, 36 strains with 4 species (*n* = 36; 0.69% prevalence) belonged to the CPS group, and 7 strains of 2 species (*n* = 7; 0.13% prevalence) to the CNS group. The most prevalent species among the MRS strains were *S. pseudintermedius* (*n* = 24; 0.46% prevalence) and *S. aureus* (*n* = 7; 0.13% prevalence). Table 4 shows the distribution of individual species of staphylococci in animal population in the observed period. The exact distribution of MRS strains in different species and groups of domestic animals is shown in Table 5. It follows from this table that MRS of all six detected species were isolated from domestic carnivores and their prevalence was the highest in these animals (*n* = 34; 1.38% prevalence). Surprisingly, three of the six MRS species were detected in rodents (*S. aureus*, *S. haemolyticus*, and *S. pseudintermedius)* (*n* = 3; 1.27% prevalence). In ruminants, pigs, solipeds, and exotic birds, MRS of one species was detected in each (*n* = 1, prevalence: 0.16%, 1.41%, 1.10%, and 0.97%, respectively). Table 6 shows the distribution of MRS isolates in different organs and organ systems. The greatest species diversity and prevalence was found in skin (*n* = 21; 4 species; 4.79% prevalence) and the respiratory system (*n* = 8; 4 species; 2.72% prevalence). All MRS strains (100%) showed resistance to oxacillin, cephalothin, cefoxitin, cefovecin, piperacillin and amoxicillin/clavulanic acid. Most of these isolates were co-resistant to enrofloxacin (93%). In contrast, the isolates showed high susceptibility to florfenicol and nitrofurantoin (100%). Susceptibility to novobiocin and rifaximin was also high (97.7% of susceptible strains). The detailed results are shown in Table 7. 

A total of 35 suspected MRS strains (according to disc diffusion method) were characterized by molecular methods, and all were positive for *mecA* gene. All MRS tested (except of two *S. aureus* isolated from cat urine and cow milk) were positive for *eno* gene encoding laminin-binding protein. Genes *cna*, *clfA*, *clfB*, and *icaA* were detected in all of seven *S. aureus* isolates, and *fnbB* was detected in six *S. aureus* isolates. None of these genes were detected in non-*S. aureus* isolates. None of MRS tested strain was positive for toxin genes *etA*, *etB* or *tsst* (see Table 8).

## 4. Discussion

Even though the time span of collection and testing of clinical samples in our study was rather short, a quite large species diversity of the isolated *Staphylococcus* strains was shown and confirmed in domestic animals. A total of 28 *Staphylococcus* species were detected. In our study, of the CPS, the major species identified was *S. pseudintermedius* (*n* = 336; 6.44% prevalence), which was predominantly found in domestic carnivores, especially dogs. The second most frequently encountered CPS was *S. aureus* (*n* = 205; 3.93% prevalence), surprisingly most often found in ruminants and solipeds, followed by *S. intermedius* (*n* = 40; 0.77% prevalence), which was also predominant in carnivores, especially dogs. Out of the CNS, the major species was *S. haemolyticus* (*n* = 68; 1.30% prevalence), most often isolated from ruminants and carnivores, followed by *S. chromogenes* (*n* = 45; 0.86% prevalence), which was most prevalent in ruminants, then followed by *S. felis* (*n* = 32; 0.61%), having a clear affinity for cats, and *S. epidermidis* (*n* = 23; 0.44% prevalence), which was most frequently isolated from clinical material from domestic carnivores, ruminants, and pigs. *S. chromogenes* has the ability to form biofilms and in veterinary medicine is also a common pathogen of the mammary gland of cattle (8.8% to 51.4% of isolated CNS strains) [45,46]. The literature sources confirmed that all the above-mentioned four CNS species may be pathogenic to animals, and some of them also to humans. *S. haemolyticus* is an opportunistic pathogen infecting debilitated human patients [18] and is often detected in veterinary laboratories in association with mastitis in cattle (12.2% to 20.3% of isolated CNS strains) [45,46]. 

Not surprisingly, most MRS isolates originate in the skin and the respiratory system of animals, which is consistent with the literature. Our findings of MRS strains of staphylococci in domestic animals confirmed that *S. aureus*, *S. pseudintermedius*, *S. intermedius*, *S. epidermidis*, and *S. haemolyticus* species may have resistance genes to methicillin, as previously reported by Votava et al. (2006) and Oreiby et al. (2019) [9,23]. In our study, the strain *S. coagulans* was also detected, which in addition to methicillin resistance also showed resistance to all beta-lactam antibiotics and to enrofloxacin. The prevalence of MRSA strains in our study was relatively low (*n* = 43; 0.82%) in comparison with 15.5% in the human clinical material of hospitalized patients [47] and the prevalence of veterinary MRSA strains isolated, for example, from clinical material from domestic carnivores (16.1%) in the Czech Republic [12], as well as from milk of cows with signs of mastitis in the Czech Republic and Egypt where the prevalence of MRSA ranged from 31.7% to 50% [24,25]. The prevalence of MRS strains of *S. pseudintermedius* (*n* = 24; 0.46%) and *S. intermedius* (*n* = 4; 0.08%) in our study is also very low in comparison with other Czech studies dealing with *S. pseudintermedius* (25%) and *S. intermedius* (50%) [12]. 

The MRS strains of *S. haemolyticus* and *S. epidermidis* are no exception, with their prevalence in our study of 0.1% (*n* = 5) and 0.04% (*n* = 2), respectively, while literature sources reported prevalence, for example in humans, of up to 45.4% for both of the above bacterial species [48]. This can be attributed to the fact that our strains were collected for 14 months only and that relatively large numbers of various materials from different animals were examined and tested, while other studies focused on narrow spectra of human patients, animals, and clinical materials such as cow’s milk samples or clinical specimens from dogs and cats. Our study also shows that the detected strains display a certain species-specificity in terms of antimicrobial resistance. 

In addition to beta-lactams, MRS *S. aureus* (MRSA) strains show 100% resistance also to tetracycline; *S. epidermidis* MRS strains also to gentamicin, tetracycline, erythromycin, and enrofloxacin; *S. haemolyticus* MRS strains also to trimethoprim/sulfamethoxazole, gentamicin, erythromycin, and enrofloxacin; MRS strains of *S. intermedius* also to gentamicin, erythromycin, clindamycin, and enrofloxacin; and MRS strains of *S. pseudintermedius* and *S. coagulans* also to enrofloxacin. 

Due to the diversity of the clinical material in our study, a diverse capture of adherence factors could be expected, as the first step of successful infection is adherence to different surfaces, depending on the site of infection. However, genes encoding MSCRAMMs were detected only in *S. aureus*, none in other staphylococci (CNS or CPS). 

The exception was the *eno* gene encoding laminin-binding protein. This gene was detected in all MRS isolated from clinical material except for two *S. aureus* isolated from cat urine and cow milk. Thus, this virulence factor was shown to be unrelated to the location of the infection, as different isolates from different sites of infection carried this gene. Moreover, other studies described a very common occurrence of the *eno* gene in both CPS and CNS (73–100%) [39,49,50]. Consistent with our results, rare occurrences of other MSCRAMM genes in CNS isolates were confirmed by other studies [50]. In contrast, in CPS including *S. intermedius*, a noticeably higher occurrence was described (*ebp*-73%, *fib*-91%, and *fnbA*-7%) [49]. In our study, neither *S. intermedius* nor *S. pseudintermedius* harbored MSCRAMM genes.

Isolates of *S. aureus* from different animals and different sites of infections (cat, pig, cow, horse, dog; urine, joint, milk, skin) were included in our study, yet all these isolates showed very similar MSCRAMM gene profiles: *eno*+ (except two isolates), *cna*+, *clfA*+, *clfB*+, and *fnbB*+ (except one isolate from cat urine).

The *icaA* gene encoding polysaccharide intercellular adhesin was detected in all MRSA isolates. The *ica* operon is considered to be the main operon responsible for biofilm formation in the *Staphylococcus* genus. However, there is not an absolute correlation between the presence or absence of *ica* genes and biofilm production [51]. In our study, the *icaA* gene was not detected in other staphylococci (non-*S. aureus*), consistent with other studies where *icaA* was only rarely detected in these strains [51,52,53]. Genes encoding *etA*, *etB*, and *tsst* toxins were not detected in our MRS isolates and also in other publications, only the rare occurrence of these genes in staphylococci of animal origin was described [51,54]. Although these virulence factors can be found in animal strains, they are generally associated with human clinical pathogens [51,55]. 

It is well known that the growth of antibiotic resistance is a global problem. Veterinary medicine undoubtedly contributes to its origin and spread due to the application of antimicrobials in water and feed, especially in livestock, and also due to the use of local antibiotics (sprays, suppositories, ointments, dusting powders), where accurate dosing of these substances is not guaranteed. It is therefore necessary to tighten up the legislation governing the use of these substances in animals and to set up a system for its control. At the same time, there is a need to expand and improve university teaching on the use of antimicrobials in the veterinary field.

## 5. Conclusions

The present study demonstrated high prevalence of some CPS and CNS species detected in animals. These strains should be taken seriously from both the epizootiological and epidemiological point of view, as they can pose health risk to both animals and humans not only in terms of potential pathogenicity but in that they can also confer resistance genes and pathogenicity factors to other veterinary and human strains of staphylococci. In terms of antimicrobial susceptibility, it has been confirmed that methicillin-resistant microorganisms are resistant to all other beta-lactam antibiotics, even in isolated veterinary strains and also to amoxicillin/clavulanic acid. All MRS strains of staphylococci isolated in the present study except for three *S. aureus* strains were resistant to enrofloxacin. Due to the occurrence of these microorganisms in farm animals and animals kept for hobby, it would be very appropriate to set up research projects aimed at the detection of oxacillin/methicillin-resistant staphylococci in these animal groups and to map their occurrence at least in Europe, because the microecosystems of animals, humans, plants, and the macroecosystem are interconnected. It is therefore necessary to emphasize the issue of correct using of antimicrobials in practice and antibiotic policy in university teaching and to create stricter legislation that would prevent the widespread use of antimicrobials in veterinary medicine, especially in livestock to reduce the emergence and spread of antimicrobial resistance.

## Figures and Tables

**Table 1:** Susceptibility table-reference values for *Staphylococcus* spp.

Antimicrobials	Antibiotics Concentration Per Disc (µg)	Diameter (mm)		
		R	S	Source
Cefoxitin (*S. aureus*, *S. lugdunensis*)	30	≤21	≥22	CLSI VET 01 S (2018)
Cefoxitin (CNS)	30	≤24	≥25	CLSI VET 01 S (2018)
Oxacillin (*S. aureus*, *S. pseudintermedius*, *Staphylococcus* spp.)	5	<20	≥20	CASFM (2018)
Amoxicillin/clavulanic acid	20/10	≤19	≥22	NCCLS (2002)
Trimethoprim/sulfamethoxazole (*Staphylococcus* spp.)	1.25/23.75	≤10	≥16	CLSI VET 01 S (2018)
Gentamicin (*S. aureus*)	10	<18	≥18	EUCAST (2020)
Gentamicin (CNS)	10	<22	≥22	EUCAST (2020)
Tetracycline (*Staphylococcus* spp.)	30	≤17	≥23	CLSI VET 01 S (2018)
Chloramphenicol (*Staphylococcus* spp.)	30	≤12	≥18	CLSI VET 01 S (2018)
Erythromycin (*Staphylococcus* spp.)	15	≤13	≥23	CLSI VET 01 S (2018)
Florfenicol (*Staphylococcus* spp.)	30	≤18	≥22	CLSI VET 01 (2013)
Clindamycin (*Staphylococcus* spp.)	2	≤14	≥21	CLSI VET 01 S (2018)
Enrofloxacin (*Staphylococcus* spp.)	5	≤16	≥23	CLSI VET 01 S (2018)
Nitrofurantoin (*Staphylococcus* spp.)	100	≤14	≥17	CLSI VET 01 S (2018)
Novobiocin	30	≤17	≥22	BD BBL (2020)
Rifaximin	40	<10	>19	BIOPHARM (2020)

S = susceptible; R = resistant; CNS = coagulase negative staphylococci.

**Table 2:** Polymerase chain reaction primers used in this study to detect virulence factor genes in methicillin-resistant staphylococci.

Gene	Primer	Nucleotide Sequence (5′–3′)	Amplicon Size	Reference
*mecA*	MECA-1	GTAGAAATGACTGAACGTCCGATAA	310	[38]
	MECA-2	CCAATTCCACATTGTTTCGGTCTAA		
*cna*	CNA-1	GTCAAGCAGTTATTAACACCAGAC	423	[39]
	CNA-2	AATCAGTAATTGCACTTTGTCCACTG		
*eno*	ENO-1	ACGTGCAGCAGCTGACT	302	[39]
	ENO-2	CAACAGCATYCTTCAGTACCTTC		
*clfA*	CLFA-1	ATTGGCGTGGCTTCAGTGCT	292	[39]
	CLFA-2	CGTTTCTTCCGTAGTTGCATTTG		
*clfB*	CLFB-1	ACATCAGTAATAGTAGGGGGCAAC	205	[39]
	CLFB-2	TTCGCACTGTTTGTGTTTGCAC		
*fib*	FIB-1	CTACAACTACAATTGCCGTCAACAG	404	[39]
	FIB-2	GCTCTTGTAAGACCATTTTCTTCAC		
*ebp*	EBP-1	CATCCAGAACCAATCGAAGAC	186	[39]
	EBP-2	CTTAACAGTTACATCATCATGTTTATCTTTG		
*bbp*	BBP-1	AACTACATCTAGTACTCAACAACAG	575	[39]
	BBP-2	ATGTGCTTGAATAACACCATCATCT		
*fnbA*	FNBA-1	CACAACCAGCAAATATAG	1362	[40]
	FNBA-2	CTGTGTGGTAATCAATGTC		
*fnbB*	FNBB-1	GTAACAGCTAATGGTCGAATTGATACT	524	[39]
	FNBB-2	CAAGTTCGATAGGAGTACTATGTTC		
*icaA*	ICAA-1	GATTATGTAATGTGCTTGGA	770	[40]
	ICAA-2	ACTACTGCTGCGTTAATAAT		
*etA*	ETA-1	CTATTTACTGTAGGAGCTAG	741	[41]
	ETA-2	ATTTATTTGATGCTCTCTAT		
*etB*	ETB-1	ACGGCTATATACATTCAATT	200	[41]
	ETB-2	TCCATCGATAATATACCTAA		
*tsst*	TSST-1	AAGCCCTTTGTTGCTTGCG	445	[42]
	TSST-2	ATCGAACTTTGGCCCATACTTT		

**Table 3:** Number and prevalence of isolated staphylococci in animals during 1 April 2019–31 May 2020.

*Staphylococcus* Species	Number of Isolated Strains (*n* =)	Prevalence (%)	*Staphylococcus* Species	Number of Isolated Strains (*n* =)	Prevalence (%)
*S. aureus*	205	3.93	Mammaliicoccus lentus *	3	0.06
*S. arlettae*	3	0.06	*S. lugdunensis*	1	0.02
*S. capitis* ssp. *capitis*	2	0.04	*S. lutrae*	3	0.06
*S. caprae*	2	0.04	*S. petrasii* ssp. petrasii *	1	0.02
*S. carnosus*	1	0.02	*S. pseudintermedius*	336	6.44
*S. caseolyticus*	1	0.02	S. coagulans *	19	0.36
*S. chromogenes*	45	0.86	*S. schleiferi* ssp. schleiferi *	2	0.04
*S. cohnii* ssp. *cohnii*	1	0.02	Mammaliicoccus sciuri *	18	0.34
*S. delphini*	3	0.06	*S. simulans*	11	0.21
*S. epidermidis*	23	0.44	*S. succinus* ssp. succinus *	2	0.04
*S. equorum*	7	0.13	Mammaliicoccus vitulinus *	1	0.02
*S. felis*	32	0.61	*S. warneri*	2	0.04
*S. haemolyticus*	68	1.30	*S. xylosus*	16	0.31
*S. hyicus*	6	0.11			
*S. intermedius*	40	0.77	Total	854	16.37

* Nomenclature changes byMadhaiyan et al. (2020) [44].

**Table 4:** Total number of staphylococci strains (by species) isolated from animals during 1 April 2019–31 May 2020.

*Staphylococcus* spp.	*S. aureus*	*S. arlettae*	*S. capitis* ssp. *capitis*	*S. caprae*	*S. carnosus*	*S. caseolyticus*	*S. chromogenes*	*S. cohnii* ssp. *cohnii*	*S. delphini*	*S. epidermidis*	*S. equorum*	*S. felis*	*S. haemolyticus*	*S. hyicus*	*S. intermedius*	M. lentus *	*S. lugdunensis*	*S. lutrae*	*S. petrasii* ssp. petrasii *	*S. pseudintermedius*	S. coagulans *	*S. schleiferi* ssp. schleiferi *	M. sciuri *	*S. simulans*	*S. succinus* ssp. succinus *	M. vitulinus *	*S. warneri*	*S. xylosus*	*Total*
Animal (Group)																													
Domestic carnivores	6	0	2	0	1	0	3	0	1	8	0	32	14	0	38	0	0	2	1	333	11	2	2	1	0	0	1	0	458
(dogs and cats)																													
Ruminants	171	3	0	2	0	1	40	1	0	5	1	0	42	1	0	2	0	1	0	0	3	0	10	9	2	1	1	12	308
Pigs	6	0	0	0	0	0	0	0	0	4	0	0	0	5	0	0	0	0	0	0	2	0	0	1	0	0	0	0	18
Solipeds	12	0	0	0	0	0	2	0	2	0	6	0	0	0	2	1	1	0	0	1	2	0	0	0	0	0	0	0	29
Birds	4	0	0	0	0	0	0	0	0	1	0	0	0	0	0	0	0	0	0	0	0	0	0	0	0	0	0	3	8
Exotic mammals	1	0	0	0	0	0	0	0	0	2	0	0	0	0	0	0	0	0	0	0	1	0	0	0	0	0	0	0	4
Exotic birds	1	0	0	0	0	0	0	0	0	3	0	0	0	0	0	0	0	0	0	0	0	0	0	0	0	0	0	1	5
Rodents	4	0	0	0	0	0	0	0	0	0	0	0	12	0	0	0	0	0	0	2	0	0	6	0	0	0	0	0	24
Total	205	3	2	2	1	1	45	1	3	23	7	32	68	6	40	3	1	3	1	336	19	2	18	11	2	1	2	16	854

No staphylococci were isolated from reptiles, insects (bee), and fishes. * Nomenclature changes by Madhaiyan et al. (2020) [44].

**Table 5:** Number and prevalence (%) of oxacillin-resistant staphylococci strains isolated from domestic animals during 1 April 2019–31 May 2020.

*Staphylococcus* Species	*S. aureus*	*S. epidermidis*	*S. haemolyticus*	*S. intermedius*	*S. pseudintermedius*	*S. coagulans*	Total	Number of Samples
Animal (Group)								
Domestic carnivores (dogs and cats)	1 (0.04)	1 (0.04)	4 (0.16)	4 (0.16)	23 (0.93)	1 (0.04)	34 (1.38)	2471
Ruminants	3 (0.16)	0	0	0	0	0	3 (0.16)	1836
Pigs	1 (1.41)	0	0	0	0	0	1 (1.41)	71
Solipeds	1 (1.10)	0	0	0	0	0	1 (1.10)	91
Birds	0	0	0	0	0	0	0	242
Exotic mammals	0	0	0	0	0	0	0	84
Exotic birds	0	1 (0.97)	0	0	0	0	1 (0.97)	103
Exotic reptiles	0	0	0	0	0	0	0	46
Fish	0	0	0	0	0	0	0	35
Insects(bees)	0	0	0	0	0	0	0	3
Rodents	1 (0.42)	0	1 (0.42)	0	1 (0.42)	0	3 (1.27)	236
Total	7 (0.13)	2 (0.04)	5 (0.10)	4 (0.08)	24 (0.46)	1 (0.02)	43 (0.82)	5218

**Table 6:** Number and prevalence (%) of oxacillin-resistant staphylococci strains isolated from organs of domestic animals during 1 April 2019–31 May 2020.

*Staphylococcus* Species	*S. aureus*	*S. epidermidis*	*S. haemolyticus*	*S. intermedius*	*S. pseudintermedius*	*S. coagulans*	Total	Number of Samples
Organ (Apparatus)								
Ear	0	0	0	0	4 (0.79)	0	4 (0.79)	507
Eye	0	0	0	0	2 (1.12)	0	2 (1.12)	179
Skin	2 (0.46)	0	0	4 (0.91)	14 (3.20)	1 (0.23)	21 (4.79)	438
Respiratory	1 (0.34)	1 (0.34)	4 (1.36)	0	2 (0.68)	0	8 (2.72)	294
Digestive	0	0	0	0	2 (0.10)	0	2 (0.10)	1983
Mammary gland	3 (0.19)	0	0	0	0	0	3 (0.19)	1576
Urogenital	0	1 (0.49)	1 (0.49)	0	0	0	2 (0.97)	206
Musculoskeletal	1 (3.33)	0	0	0	0	0	1 (3.33)	30
Lymphatic	0	0	0	0	0	0	0	2
Circulation	0	0	0	0	0	0	0	3
Nervous	0	0	0	0	0	0	0	0
Total	7 (0.13)	2 (0.04)	5 (0.10)	4 (0.08)	24 (0.46)	1 (0.02)	43 (0.82)	5218

**Table 7:** Susceptibility of oxacillin-resistant strains of staphylococci isolated from domestic animals during 1 April 2019–31 May 2020 to antimicrobials (number of susceptible/examined and percentage of susceptible).

*Staphylococcus* Species	*S. aureus*	*S. epidermidis*	*S. haemolyticus*	*S. intermedius*	*S. pseudintermedius*	*S. coagulans*	Total
Antimicrobials							
Rifaximin	7/7 (100%)	1/2 (50%)	5/5 (100%)	4/4 (100%)	24/24 (100%)	1/1 (100%)	42/43 (97.7%)
Trimethoprim/ sulphamethoxazole	6/7 (85.7%)	1/2 (50%)	0/5 (0%)	2/4 (50.0%)	11/24 (45.8%)	1/1 (100%)	21/43 (48.8%)
Gentamicin	3/7 (42.9%)	0/2 (0%)	0/5 (0%)	0/4 (0%)	5/24 (20.8%)	1/1 (100%)	9/43 (20.9%)
Tetracycline	0/7 (0%)	0/2 (0%)	2/5 (40.0%)	1/4 (25.0%)	4/24 (16.7%)	1/1 (100%)	8/43 (18.6%)
Chloramphenicol	7/7 (100%)	2/2 (100%)	5/5 (100%)	2/4 (50.0%)	17/24 (70.8%)	1/1 (100%)	34/43 (79.1%)
Florfenicol	7/7 (100%)	2/2 (100%)	5/5 (100%)	4/4 (100%)	24/24 (100%)	1/1 (100%)	43/43 (100%)
Erythromycin	6/7 (85.7%)	0/2 (0%)	0/5 (0%)	0/4 (0%)	2/24 (8.3%)	1/1 (100%)	9/43 (20.9%)
Clindamycin	5/7 (71.4%)	1/2 (50%)	2/5 (40.0%)	0/4 (0%)	2/24 (8.3%)	1/1 (100%)	11/43 (25.6%)
Enrofloxacin	3/7 (42.9%)	0/2 (0%)	0/5 (0%)	0/4 (0%)	0/24 (0%)	0/1 (0%)	3/43 (7.0%)
Novobiocin	7/7 (100%)	2/2 (100%)	4/5 (80.0%)	4/4 (100%)	24/24 (100%)	1/1 (100%)	42/43 (97.7%)
Nitrofurantoin	7/7 (100%)	2/2 (100%)	5/5 (100%)	4/4 (100%)	24/24 (100%)	1/1 (100%)	43/43 (100%)

**Table 8:** Occurrence of virulence factor genes in methicillin-resistant *Staphylococcus aureus* (*n* = 7).

Animal	Matter	*mecA*	*cna*	*eno*	*clfA*	*clfB*	*fnbB*	*icaA*
Cat	urine	+	+	-	+	+	-	+
Pig	joint	+	+	+	+	+	+	+
Cat	skin	+	+	+	+	+	+	+
Cow	milk	+	+	+	+	+	+	+
Cow	milk	+	+	-	+	+	+	+
Horse	skin	+	+	+	+	+	+	+
Dog	skin	+	+	+	+	+	+	+

*mecA*—gene encoding methicillin resistance, *can*—collagen-binding protein gene, *eno*—laminin-binding protein gene, *clfA* and *clfB*—genes encoding clumping factors, *fib*—fibrinogen-binding protein gene, *ebp*—elastin-binding protein gene, *bbp*—bone sialoprotein-binding protein gene, *fnbA* and *fnbB*—genes encoding fibronectin-binding proteins, *icaA*—polysaccharide intercellular adhesin gene, *etA* and *etB*—exfoliative toxin genes, *tsst*—toxic shock syndrome toxin gene.

## Data Availability

All data supporting reported results of this work are archived in the personal database of the first author (Bzdil, J.) and partial data concerning the genomic characterization of bacterial strains are stored in the database of the Veterinary Research Institute Brno (Czech Republic).
